# Sterile Insect Technique Programme against Mediterranean Fruit Fly in the Valencian Community (Spain)

**DOI:** 10.3390/insects12050415

**Published:** 2021-05-06

**Authors:** Ignacio Plá, Jaime García de Oteyza, Carlos Tur, Miguel Ángel Martínez, María Carmen Laurín, Ester Alonso, Marta Martínez, Ángel Martín, Román Sanchis, María Carmen Navarro, María Teresa Navarro, Rafael Argilés, Marta Briasco, Óscar Dembilio, Vicente Dalmau

**Affiliations:** 1 Empresa de Transformación Agraria S.A., S.M.E., M.P. (TRAGSA), Avenida de la Industria 26, 46980 Paterna, Spain; jgarciad@tragsa.es (J.G.d.O.); ctur@tragsa.es (C.T.); mmarti17@tragsa.es (M.Á.M.); mlaurin@tragsa.es (M.C.L.); ealonso4@tragsa.es (E.A.); mmarti79@tragsa.es (M.M.); amartin2@tragsa.es (Á.M.); rsanchis@tragsa.es (R.S.); mnavarr7@tragsa.es (M.C.N.); mnavar11@tragsa.es (M.T.N.); rargiles@hotmail.com (R.A.); martabrsc@gmail.com (M.B.); odembili@tragsa.es (Ó.D.); 2 Escuela de Doctorado, Universidad Católica de Valencia San Vicente Mártir, 46001 Valencia, Spain; 3 Servicio de Sanidad Vegetal, Conselleria de Agricultura, Desarrollo Rural, Emergencia Climática y Transición Ecológica, Ctra. Alicante-Valencia, s/n Apdo. Correos 125, 46460 Silla, Spain

**Keywords:** *Ceratitis capitata*, mass-rearing, fly emergence and release facility, irradiation, integrated pest management, autocidal control

## Abstract

**Simple Summary:**

The Mediterranean fruit fly, *Ceratitis capitata* (Wied.), is one of the most destructive fruit pests in the world. In the Valencian Community, it has become a key pest affecting many fruits, but mainly citrus, the most important crop cultivated. Traditionally, control of *C. capitata* has been based on the use of insecticides. In line with the trend in recent years to minimize the use of insecticides and promote environment-friendly techniques, in 2007 the Department of Agriculture of Valencia implemented an area-wide integrated pest management (AW-IPM) programme for the suppression of *C. capitata*, in which the sterile insect technique (SIT) is the primary method of application integrated with other suppression methods. As a result, there has been a large reduction in the aerial spraying of insecticides as well as a growth trend in exports of citrus and fresh fruits from the Valencian Community in recent years.

**Abstract:**

The Mediterranean fruit fly, *Ceratitis capitata* (Wied.), is an endemic pest in fruit-growing areas of the Spanish Mediterranean coast. In the Valencian Community, it represents a serious problem in the cultivation of citrus and numerous species of fruit, such as peach, cherry, apricot, persimmon, etc. For over 50 years, the Department of Agriculture of Valencia has led, promoted, and carried out a *C. capitata* control programme to protect crops, especially citrus fruits, because this community is the largest national producer and the leading region for fresh citrus exports in the world. Traditionally, pest control has been based on the use of insecticides. However, a reduction of more than 90% of a target wild population was achieved in the frame of a pilot integrated pest management (IPM) project based on the sterile insect technique (SIT), which was implemented from 2003 to 2006. Based on this successful result, in 2007 the Department of Agriculture of Valencia initiated an area-wide integrated pest management (AW-IPM) programme for the suppression of *C. capitata*, using the SIT as the primary control method. Complementary activities are implemented periodically in hotspots and during different time periods depending on the pest population dynamics. As a result, there has been a reduction of more than 90% in the use of insecticides by aerial means to control *C. capitata*, as well as a growth trend in exports of citrus and fresh fruits from the Valencian Community in recent years. This paper provides a historical review of the Valencian programme and briefly describes how technological innovations and decision-making tools have contributed to programme efficiency.

## 1. Introduction

The Mediterranean fruit fly, *Ceratitis capitata* (Wied.) (Diptera: Tephritidae), is known as one of the most damaging pests to agriculture worldwide and is subject to strict quarantine measures in many countries where it is not officially present [1]. It can infest more than 330 plant species, demonstrating a high degree of adaptation to a wide range of climatic conditions [2]. It is currently considered a key pest of economic importance for Spanish agriculture, with citrus fruits being the crop most affected by its populations [3].

In the Valencian Community (Spain), *C. capitata* not only affects citrus fruits (both late and early species and varieties) but also other fruits that ripen in late spring, during the summer, and early autumn, with some being even more seriously affected than citrus in certain areas [4]. This pest could potentially negatively affect more than 1 million tons of citrus and other fruits in this region. To overcome this situation, an area-wide integrated pest management (AW-IPM) programme has been implemented, including the following actions:

- Installation of a monitoring trapping network
- Mass-rearing, sterilization, and handling of *C. capitata* genetic sexing strain (GSS)
- Release of sterile *C. capitata* males
- Establishment of strategic areas for other control methods, such as mass-trapping, control of isolated *C. capitata* hosts, etc.
- Insecticide application: aerial and/or ground application in hotspots on a collective basis.

These actions complement one another and are integrated and well-coordinated, with the sterile insect technique (SIT) being the central axis of the AW-IPM programme [5,6].

The SIT is an autocidal control method that is based on the mass-rearing, radiation-based sterilization, and release of the same species that is targeted for population control. In the case of *C. capitata*, a GSS is available (VIENNA 8), which carries two selectable markers (the *white pupae* (*wp*) and *temperature sensitive lethal* (*tsl*) genes), allowing the release of only males. After being sterilized by irradiation, sterile males are released in the areas with affected crops and mate with wild females, which, as a result, lay eggs that are not viable and consequently produce no offspring [7]. Over generations, this results in a decline of the target wild populations, thus keeping the pest under control, i.e., below a pre-determined economic damage threshold. This method of control has been used to manage insect pest populations since the 1960s. E.F. Knipling conceived the SIT approach to control populations of insect species, and the concept was first successfully tested against the New World screwworm fly (*Cochliomyia hominivorax* (Coquerel)) in 1957 [8]. Following the successful application of SIT against *C. hominivorax*, the potential of SIT as a tool for suppression, prevention, containment, or local eradication was tested on other insects of economic importance, such as *C. capitata*, other tephritid species, tsetse flies, and moths [9,10,11].

In the case of the Mediterranean fruit fly, Mexico and the USA have been using SIT for prevention; Peru, Argentina, the Dominican Republic, and Chile for local eradication; Israel [12], South Africa, and Croatia for suppression; and Guatemala for containment [7]. In addition, the SIT has been successfully applied against other fruit flies, such as the melon fly (*Zeugodacus cucurbitae*) on the Okinawa Islands of southern Japan, the *Anastrepha* species in northern Mexico, and the Queensland fruit fly (*Bactrocera tryoni*) in Australia [11,13].

Given the successful application of the SIT for the control of agricultural and livestock pests in other countries, the Valencian authorities initiated a large scale operational AW-IPM programme in 2007 with the SIT as a major component to suppress Mediterranean fruit fly populations in the region [14]. This programme has been implemented by the state-owned company Empresa de Transformación Agraria S.A. (Grupo TRAGSA, Valencia, Spain), a leading company in the execution of environment-friendly projects in Spain. The “Bioplanta” mass-rearing facility (MRF) was designed and built by this company with the assistance of the Joint Food and Agriculture Organization of the United Nations (FAO) and the International Atomic Energy Agency (IAEA) Centre (previously called Division) of Nuclear Techniques in Food and Agriculture (hereinafter called Joint FAO/IAEA Centre). It is currently one of the largest facilities in the world, with a maximum production capacity of 500 million sterile pupae per week. VIENNA 8 GSS-Valencia pupae from a thermal colony are sterilized by exposure to 100 Gy under a continuous-wave E-beam accelerator (RHODOTRON^®^ TT200–Ion Beam Applications S.A.). After sterilization, pupae are transported and adults emerged at the “Evolucionario” packing and release center (PRC), also designed and built by Grupo TRAGSA in 2003. Upon reaching sexual maturity, 1-day prior sterilized flies are released by air over citrus target areas covering a total of more than 140,000 hectares. To protect this area, the programme since its beginning has produced from 13,000 to 15,000 million sterile male pupae per year. The total cost of the entire AW-IPM programme is about 9.5 million Euros per year, mainly financed by the Department of Agriculture of Valencia and the Spanish Ministry of Agriculture.

We herein present the main activities and achievements of the programme during the last 13 years, including the mass-production of the GSS of the Mediterranean fruit fly, the irradiation process, the handling of sterile insects at the fly emergence and release facility, the aerial releases, quality control at all steps, and post-release monitoring activities. We also present the socio-economic impact of the programme to the agricultural industry and discuss the programme’s main challenges and future goals.

## 2. Production of Sterile Male Pupae

Sterile male pupae of *C. capitata* VIENNA 8 GSS-Valencia are produced at the MRF located in Caudete de las Fuentes (Valencia). The VIENNA 8 GSS was originally developed at the Insect Pest Control Laboratory of the Joint FAO/IAEA Centre, Seibersdorf, Austria [15]. In 2015, the strain was refreshed with Valencia local wild population [16], and as a consequence, the Vienna 8 GSS-Valencia strain was obtained. This strain has two selectable markers which allow male-only production, thus enhancing the efficiency and cost-effectiveness of SIT because: (a) sterile males are not engaged in mating with sterile females [13]; (b) sterile females are not released and thus do not oviposit in fruits and vegetables; and (c) females are not mass-produced or released, reducing production costs. The two selectable markers that allow male-only production are (1) *white pupae* (*wp*): female pupae are white in color while male pupae are brown (wild color), and (2) *temperature sensitive lethal* (*tsl*): this second characteristic allows the selective removal of females by exposing the embryos to elevated temperatures after 24–36 h of incubation. In addition, this strain shows increased fecundity, a higher temperature threshold to eliminate the females (35 °C compared to 34 °C observed for the original VIENNA 8 strain), and faster female larval development, reducing by 1 day the time needed for larval collection.

**Figure 1:**
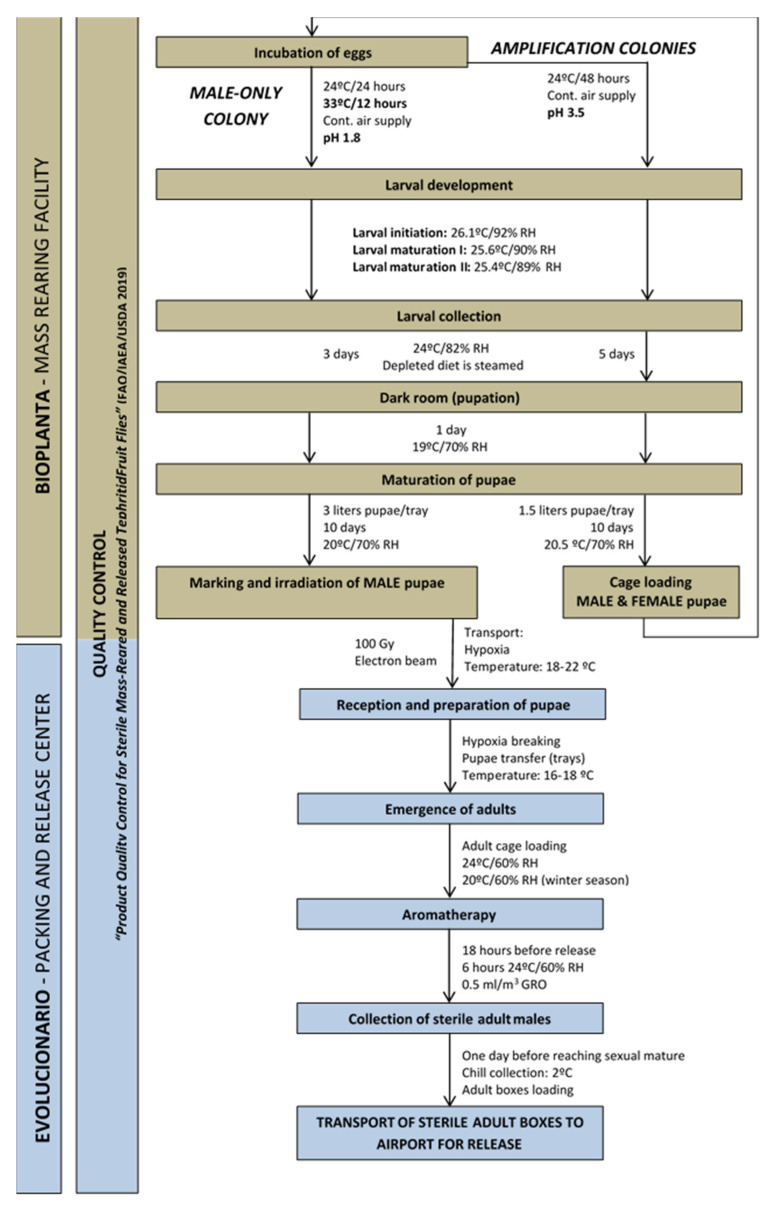
Flow chart of the activities conducted at the Mass-Rearing Facility & Packing and Release Center.

When mass-rearing a GSS, a filter colony system must be implemented. This filter colony is composed of a small number of individuals ensuring that males emerge from brown pupae and females emerge from white pupae, and that any detected recombinant individuals are removed [17,18,19]. A progressive increase in the number of produced insects is achieved by amplification colonies (Figure 1), which are constantly renewed for obtaining young egg-laying females. The increased production of eggs by amplification colonies, currently two in our programme, allows the establishment of the “thermal colony”, in which females are eliminated by heat-treatment of the eggs.

Eggs of amplification and thermal colonies are seeded on a larval rearing medium (larval diet), where larvae feed and develop. Fully developed third-instar larvae jump out of the larval diet trays and pupate in a wheat bran substrate. The whole rearing process (egg to adult) takes about 25 ± 2 days.

One day before adult emergence, males are sterilized by exposure to ionizing radiation under a continuous-wave E-beam accelerator. Once sterilized, pupae are sent to the PRC located in Moncada (Valencia). Both amplification and thermal colonies are recharged daily, and in this way, the production cycle is continuous. At any time, all the colonies within the MRF are being reared simultaneously.

### 2.1. Incubation of Eggs

Eggs obtained from mature females are loaded daily into incubators, which are thermostatic baths with a constant air supply. They are “air bubbled” there for 48 h at 24 °C, which is the time required for completion of embryonic development at that temperature. The continuous supply of air prevents a decrease in the dissolved oxygen level within the incubation medium (detrimental to the development of the embryos) and keeps the eggs in suspension. The incubation temperature for the eggs of the thermal colony is increased, and 24 h-old embryos are exposed to a temperature of 33 °C for 12 h. This treatment will not kill all the females, as the threshold of the VIENNA 8 GSS-Valencia strain is 35 °C. However, we have shown that total productivity (egg–pupae) can be increased by 1% if white-pupae female survivors of the heat treatment are removed at the pupal stage by color sorting. This is only performed on the last larvae collection day and its cost-effectiveness has been confirmed (data not shown). All of this allows us to obtain only male pupae, which leads to a reduction in production costs and significantly increases the effectiveness of the SIT in the field.

For the incubation of the embryos of amplification colonies, the pH of the medium is set at 3.5, while it is set at 1.8 for the thermal colony. This reduction of the pH value prevents, to a certain extent, the proliferation of microorganisms that might jeopardize the productivity of the rearing process.

The overall objective of this step is to obtain fully developed embryos, with a low percentage of larvae at seeding time for amplification colonies (0.5–5%) and a medium percentage for the thermal colony (10–35%). In the latter case, the temperature at which eggs are held for the first 24 h of incubation might be adjusted as required.

### 2.2. Preparation of Larval Diet

The composition of the artificial larval diet is fundamental for efficient and cost-effective mass-rearing. In each rearing facility, the formulation varies depending on the cost and availability of the ingredients available in the local market. The standard larval diet includes the following three components: bulking agent and water as support medium (these two components represent about 75% of the formulation), sugar and inactive yeast as nutrients (about 22% of the formulation), and preservatives to prevent the growth of microorganisms that may affect larval development. The larval diet in the MRF consists of sugar beet pellets (11.03%) as bulking agent, water (66.68%), sugar and inactive brewer’s yeast (11.16 and 9.87%, respectively) as nutrients, and hydrochloric acid, sodium benzoate, and nipagin (0.60, 0.30 and 0.36%, respectively) as preservatives. The ingredients are introduced into a mixer with a maximum capacity of 2.1 tons. Once the homogeneous mixture is ready, the diet is adjusted in humidity and pH, which varies if eggs are going to be seeded on trays for the amplification or thermal colonies (3.5 and 3.8, respectively) with 4.5 kg of larval diet at a rate of 17 viable eggs per gram of diet.

### 2.3. Larval Development

*Ceratitis capitata* larvae pass through three stages (instars) to complete their development. In addition to the larval diet, efficient larval rearing requires stable and suitable environmental conditions for each developmental stage.

Experimental work has shown that a higher yield from the thermal colony is obtained when the larval diet is kept under the same conditions as the diet for the amplification colonies. The different larval development stages require different environmental conditions and are therefore maintained in different rooms, i.e., Larval Initiation (2 days at 26.1 °C and 92% RH), Larval Maturation I (1 day at 25.6 °C and 90% RH), and Larval Maturation II (3.5 days at 25.4 °C and 89% RH). The lower temperatures of the rooms extend the larval maturation period by 1 day.

### 2.4. Larval Collection

Mature third-instar larvae leave the diet in which they have developed, i.e., they move to the sides of the trays by small and sudden hops, and finally jump out of the trays in search of a suitable place for pupation. The racks with the larval diet trays are transferred to the collection room (shared by both production lines) and placed over a wheat bran layer as pupation substrate. Depending on the number of larvae jumping out, the collection trays may be replaced up to 3 times per day. The racks remain in the room for 3 days in the case of the thermal colony and 5 days for the amplification colonies. After the end of this period, the remaining diet is steamed at 85 °C to eliminate larvae that did not complete their development. The residual diet is sent to local sheep farms after the Animal Production Department of the Polytechnic University of Valencia has certified the diet as suitable animal feed.

### 2.5. Maturation of Pupae

Larval collection trays are moved to a dark room (19 °C, 70% RH) for 12–24 h for completion of pupation. Subsequently, the wheat bran is removed by sieving, and the clean pupae are placed in net trays (60 × 40 × 3.5 cm) to complete their development in pupae maturation rooms. Each tray receives 3 liters of pupae from the thermal colony and is kept for 10 days at 20 °C and 70% RH; in the case of amplification colonies, each tray is filled with 1.5 L of pupae and is kept for 10 days at 20.5 °C and 70% RH.

### 2.6. Sterilization

Pupae of the thermal colonies are sent to be sterilized by exposure to irradiation under a continuous-wave electron beam accelerator one day before completing their development.

The used irradiation method delivers a good homogeneous dose with only 10% variability when sample height does not exceed 5 cm and the treatment with 100 Gy results in an average sterility of 98.87 ± 0.55%. 

Within the same container, the variability of the sterility obtained is less than 0.3%, which has allowed an increase in the height of the pupae transport containers by 1 cm, achieving a 20% cost savings in the irradiation process. Before irradiation, pupae are marked with a fluorescent dye (RadGlo JST 17, Radiant Color, Belgium). This pigment enables the subsequent identification of sterile males in the laboratory when they are trapped in the monitoring traps that are deployed in the field. The pupae are marked in a rotating drum that contains a given amount of fluorescent dye, so that emerging adults are impregnated with the dye, which is easily distinguished under ultraviolet light. The amount of fluorescent pigment used is 1 g per L of pupae for the local programme, while 1.5 g per L is recommended when pupae are exported [20].

The quality control of pre-irradiation insects is performed following the USDA/FAO/IAEA “Product Quality Control for Sterile Mass-Reared and Released Tephritid Fruit Flies” [21].

### 2.7. Oviposition

Pupae from the amplification colonies are used to replenish adult cages. One day before emergence, they are loaded into cages where they have access to food (4:1 sugar:autolyzed yeast) and water. These cages are assembled daily and, depending on the colony they belong to, the female to male ratio as well as the number of insects inside them vary, ranging from 1:1.1 for the first amplification colony to 1.5:1 for the second, keeping the adult density under 1 adult per 5 cm^2^. Twenty-five percent of the males in the cage are 2 days younger than the females, and the rest of the males; this increases total egg production by 8% (Antonio Polido, personal communication). Immature and mature adult cages have the same temperature and relative humidity requirements and are therefore kept in the same room. When adults emerge, they remain in pre-oviposition for 3 days until they reach sexual maturity. At this point, females start laying eggs through the mesh of the cages, which are recovered by gravity. As long as the rate of oviposited eggs is high, these cages will remain in production (12 days). Eggs are collected 2 times per day. After collection, they are cleaned and transferred to the incubators, and the production cycle is completed.

An important factor for achieving high egg production is to maintain good environmental conditions in terms of low load of adult scent in the oviposition room. This can be achieved by having low numbers of adults per cubic meter or by increasing the air exchange of the room. Under extreme conditions, productivity might drop up to 40% of the expected value (data not shown).

## 3. Development of Pupae and Emergence of Sterile Adults

Irradiated pupae are shipped from the MRF to the PRC in Moncada (Valencia). To avoid loss of biological quality of sterile males, the pupae are transported at a temperature between 18 and 22 °C and under hypoxia conditions (very low oxygen level) in sealed containers that prevent the entrance of oxygen. If these conditions are not maintained, there could be a significant increase in the temperature and humidity levels, which could reduce the quality and even cause the death of the pupae. With proper control of temperature and hypoxia, pupae development slows down significantly during transport from the MRF to the PRC.

### 3.1. Reception and Preparation of Pupae

As soon as the containers of pupae arrive at the PRC, the seals are broken and the pupae are transferred to trays that are placed in carts and taken to a room of variable temperature depending on the pace of development required.

### 3.2. Emergence of Adults

When first emergence is noticed on the pupal tray, it means the pupae are starting to emerge and must be loaded into adult cages. For this, they are placed in small containers, which are introduced into the cages. Each cage consists of 20 tubes of muslin cloth and a maximum capacity of half a million pupae per cage. A bar with sugar for feeding flies is placed inside each tube. The mesh walls allow permanent air exchange with the room, avoiding high relative humidity within the cage due to insect breathing that could affect their flight ability.

Cages are kept in rooms at 24 °C and 60% RH. The temperature is adjusted to 20 °C during the winter season for better acclimation to outdoor conditions [22]. Adults develop under these conditions until they are released in the field, 48 h after achieving 50% of emerged pupae. At noon the day before their release, mature insects are exposed for 6 h to aromatherapy treatment based on ginger root oil (GRO) essence with 40% zingibrene [23,24]. This oil is applied by aerial diffusion in a room at 24 °C and 60% humidity, at a rate of 0.5 mL per cubic meter of available space. To achieve a homogeneous distribution, the total dose of ginger oil is distributed in 8 pieces of sponge hung from 6 fans distributed throughout the room. Under Valencia programme conditions, sterile males that receive the aromatherapy with GRO achieve an increase in mating performance of up to 20% compared to those that do not receive this treatment (data not shown). Similar results are expected to be achieved under field conditions.

### 3.3. Collection of Adults

The day sterile males are going to be released, the cages that hold them are placed in a cold room at 2 °C for 30 min, thus rendering the adults inactive. Adults are collected by placing the cages in an upright position and by shaking the walls. Then they are placed in boxes to be transported to the aircraft for release. The boxes are transported to the airport in a refrigerated van.

Each cage contains half a million pupae, of which about 450,000 emerge as adults (approx. 90%). Since the capacity of the release machine is 12 million insects, around 25 cages are needed per flight.

To avoid condensation in the aircraft boxes, it is very important to maintain continuous air flow throughout the process of emptying cages and during transportation to the airport. If this is not achieved, the wings of the adult flies might get wet, thus reducing their flight ability.

The process followed is in general in line with the Joint FAO/IAEA “Guideline for packing, shipping, holding and release of sterile flies in area-wide fruit fly control programmes” [20].

### 3.4. Quality Control

**Figure 2:**
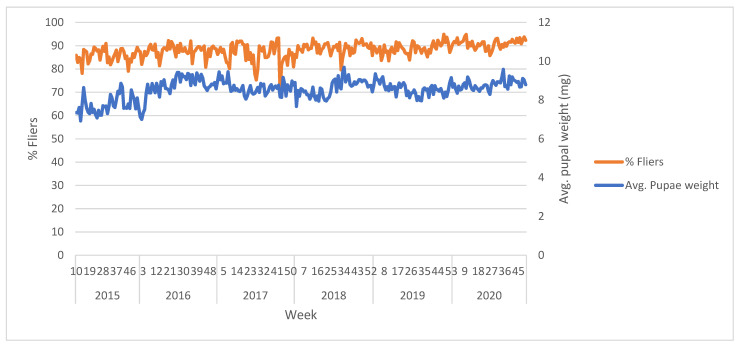
Quality control data: average weekly values for pupae weight and flight ability of the VIENNA 8 GSS-Valencia (samples from PRC).

**Figure 3:**
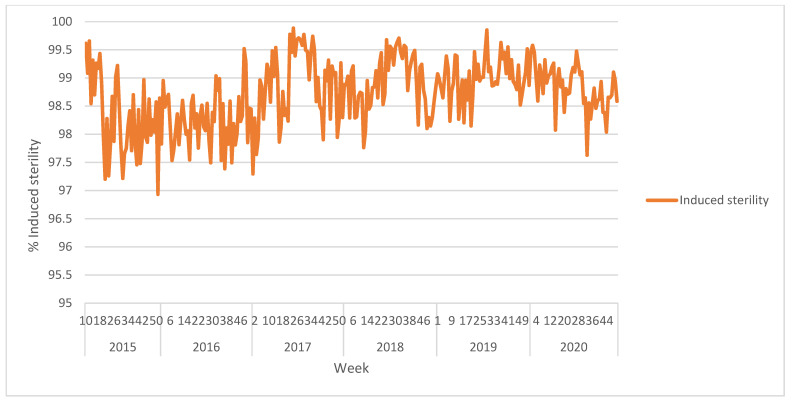
Induced sterility levels achieved after 100 Gy β-ray exposure of the VIENNA 8 GSS-Valencia brown (male) pupae. Tests carried out with wild-type females.

Pupae received by the PRC as well as the emerged adults are subjected to quality control tests listed in “Product Quality Control for Sterile Mass-Reared and Released Tephritid Fruit Flies, Version 7.0.“ [21] (Figure 2 and Figure 3).

These tests determine the effect of overcrowded holding conditions and the release process on the quality of the insects. For this purpose, a cylindrical wire mesh container 9 cm high and 8 cm in diameter is settled in the hopper of the airplane to collect a sample of the flies to be released. When the flight is over, the container is transferred into a methacrylate emergence box to prevent the flies from escaping. This box is taken to the quality control laboratory and three 5 mL samples are used for the flight ability test, and six more for the survival under stress test.

### 3.5. Aerial Release of Sterile Insects

**Figure 4:**
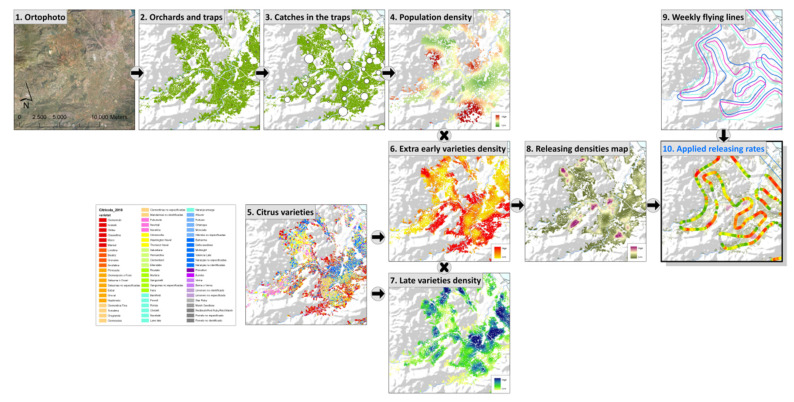
Procedure for obtaining the risk map (8) by combining the information from the monitoring network (4) and the distribution of susceptible citrus varieties in a target area (6–7). The variable-rate release path (10) to be followed by the aircraft during the release is defined after combining the risk map (8) and the predetermined release route (9).

The release of sterile flies is carried out by aircraft equipped with a chilled adult release system. The machine is temperature-controlled and can maintain proper cooling and ventilation between the flies throughout the flight. This avoids overheating and condensation among the insects. Depending on the density of the wild population in the area flown over, the number of sterile insects released is adjusted by the release software. At the bottom of the machine, an auger releases the sterile males as the flight progresses along a previously defined route. The number of flies released depends on the rotation speed of the auger. Depending on the density of the wild population in the release area, the number of sterile insects to be released is adjusted. The release rate is determined on the basis of a “risk map” that is developed using the information from the trap monitoring network and the distribution of susceptible crop varieties (Figure 4). The system is fully autonomous and does not require any operator intervention during flight. The control of releases is performed by a differential global positioning system (DGPS) installed in the aircraft. The areas where the sterile flies have been released as well as the expected release routes are checked daily.

### 3.6. Monitoring Fruit Fly Populations

A monitoring network consisting of more than 1200 traps (Nadel^®^ with trimedlure and Thepri trap^®^ with female biased attractants) distributed throughout the Valencian Community citrus area has been set up for the assessment of Mediterranean fruit fly population dynamics in the field.

The traps are inspected once a week and trapped males are examined under ultraviolet light in the PRC laboratories and identified as sterile or wild. All traps are georeferenced, and each field inspector uses a smartphone to monitor and control their trapping route. Trapping data are uploaded and sent daily via the internet to the database.

### 3.7. Data Analysis and Geographical Information System (GIS)

Wild and sterile population levels are estimated through the catches in each of the monitoring traps. Citrus information is available in a GIS, from which the variety of citrus is known in each of the plots of the Valencian Community. This allows us to analyze information spatially in a GIS. Maps can be developed that show the dynamics of the Mediterranean fruit fly population, sterile to wild male ratios, the rate of induced sterility, and the abundance of susceptible citrus varieties each season. In addition, the weekly risk map determines the rate of sterile flies to be released in each release zone and the release routes.

## 4. Research and Development Activities

In 2017, the Department of Agriculture of Valencia decided to include a research and development component in the programme, under the scientific management of the Instituto Valenciano de Investigaciones Agrarias (IVIA), with the main objective of enhancing the Valencian Community SIT AW-IPM Programme against *C. capitata*. For this purpose, the feasibility and cost-effectiveness of new strategies/tools and equipment, as well as their impact on the performance and occupational health and safety of workers, are being studied.

The performance of released sterile males is a key factor for any SIT programme, and there are ongoing research efforts focusing on the following:

- The evaluation of new nutritional components for larval and adult diets
- The role of microbiota of larvae and adults in mass-rearing efficiency and the performance of sterile males in the field.

Regarding the use of biological control agents against *C. capitata*, entomopathogenic fungi (EF) and parasitoids are being tested. Interestingly, it has been proposed that sterile flies can be used as vectors of entomopathogenic fungal spores in the frame of AW-IPM projects. Different trials are currently in the design phase to evaluate *Paecilomyces fumosoroseus*, *Beauveria bassiana*, and two strains of *Metarhizium brunneum* against *C. capitata*. The first phase of the trials will have the objective of determining the degree of pathogenicity and virulence of each of the strains, selecting one at an adequate inoculation dose to achieve the objective of transmitting conidia to wild females during mating and to wild males during the formation of leks (male aggregation). The second phase of the trials will be aimed at evaluating the horizontal transmission rate of inoculated sterile males to the wild population, as well as their sexual competitiveness compared to wild males under laboratory conditions. At the same time, the effects of the selected strain on the reproduction of infected wild females after mating with EF-inoculated sterile males will be evaluated to assess the effect on their offspring. Finally, with all the obtained data, trials will be designed under real field conditions to evaluate and confirm the potential of the selected strain as a biological control agent of *C. capitata*, using the sterile males of the SIT programme as vectors of the entomopathogenic fungi.

In addition, a pilot rearing system of *Diachasmimorpha longicaudata* (Hymenoptera: Braconidae) was started in January 2018 with parasitoids from the IVIA colony and reared on wild type *C. capitata* larvae. The rearing was successfully established on irradiated VIENNA 8 GSS-Valencia larvae that were derived from the colony at the MRF. A rearing manual has been developed; it describes the procedures to obtain host larvae and mass-rear the parasitoid. In full production, 20 boxes of adults with approximately 5200 individuals per box could be initially produced. With further improvements (mainly in the type of host larvae and development conditions) and taking into account the capacity of the climatic room available for this purpose, a weekly quantity of 150,000 produced parasitoids could be available for testing under field conditions.

Regarding the development of novel equipment, Grupo TRAGSA has developed a new emergence cage in collaboration with the company MAPA Technology S.L. Tests have been performed on a prototype with improved performance and handling compared to the currently used cage. The new model consists of a Euro-standard box measuring 40 × 60 × 32 cm, with the inner walls lined with fiberglass mesh. Inside the box are two sugar bars (food), an accordion-shaped plastic structure to increase the resting surface for the flies, and a tray in which 1 L of pupae is loaded, corresponding to about 55,000–65,000 sterile males. The cage also has a sliding gate on one of its sides through which a paper wick is inserted that draws water by capillarity from a tank hung on the gate. Boxes are stacked in 6-unit towers and placed on a rolling base for easy handling and maneuvering. The materials of the cage and its lightness make it very resistant and easy to handle. In addition, the possibility for automation of all processes is high, as the cages have Euro-standard sizes and allow application of the existing methodologies, i.e., from loading with pupae to removing the adults, washing the cages, etc., which will significantly increase the efficiency of rearing and consequently, decrease costs in the medium term. The values of the quality parameters of the flies reared in these new cages are similar to those obtained in the currently used cages, meeting the parameters recommended in the FAO/IAEA/USDA manual “Product Quality Control for Sterile Mass-Reared and Released Tephritid Fruit Flies, Version 7.0” [21].

## 5. Economic Considerations

In Spain, more than 300,000 hectares of citrus and about 350,000 ha of non-citrus fruit trees are at risk of becoming affected by the Mediterranean fruit fly [25]. The economic and social importance of this pest species is related to the enormous importance of citrus production within the Spanish agricultural sector, and particularly in the Valencian Community. Spain is the world’s sixth-largest citrus producer (6% of world production) and the world’s largest exporter of fresh citrus (20–25%) [26]. Since the mid-1990s, the importance of this pest has increased due to the expansion of extra-early citrus species (satsumas and clementines) that are more susceptible to *C. capitata* due to their ripening in September, when population levels of the pest are usually high [27]. Pest-free countries impose very restrictive quarantine and export protocols due to the importance of this pest [11]. Therefore, countries that have *C. capitata* populations face significant direct economic losses resulting from the application of these protocols. In fact, in 2001 the losses Spanish farmers faced were estimated at 300 million Euro due to the ban on the export of Spanish clementines to the United States [5]. From that moment, quarantine methods were established and/or revisited for citrus fruits exported to countries such as Australia, Japan, and the USA. These fruits must receive post-harvest quarantine treatments to follow protocols dictated by the importing country. Currently, post-harvest quarantine treatments are based on the application of cold treatments while the fruit is in transit to the destination market. The treatment is applied by keeping the fruits at a certain temperature for a precise time that differs according to the importing country.

## 6. Conclusions

The Valencian Community is the largest national producer of citrus and the leading regional exporter of fresh citrus in the world. The SIT is a complex and management-intensive process that requires collection of baseline entomological data, precise planning, and stakeholder involvement to ensure effective implementation. Although the SIT is a highly effective technique, it is not “a stand-alone” one, as additional complementary actions are needed to enhance its efficacy, especially when wild populations are above a certain level. Before starting an AW-IPM programme with a SIT component, the choice of strategy must be carefully assessed and preparations made accordingly, biological considerations for the specific pest taken into account, considerable baseline data collected, and in-depth feasibility assessments carried out [28].

The Valencia AW-IPM suppression programme for *C. capitata* is particularly ambitious and challenging, but it remains a more realistic strategy than eradication. The initial objective of this programme was to reduce the use of insecticides in the entire Valencian Community, while keeping the wild *C. capitata* populations under control. This objective has been successfully achieved.

In the past, the control of *C. capitata* in the Valencian Community was carried out mainly using aerial and ground applications of organophosphate insecticides combined with hydrolyzed protein baits. This practice had harmful effects on human health, the environment, and beneficial organisms. Moreover, populations of *C. capitata* have developed resistance to malathion [29]. Currently, the use of insecticides within an AW-IPM programme against *C. capitata* is not the most recommended or desirable approach. For *C. capitata* control, spinosad, phosmet, and lambda cyhalothrin are the chemicals that have the least impact on natural enemies (parasitoids and predators) in the Valencian Community ecosystem and are considered the least harmful alternative to malathion [30]. Insecticide treatments (aerial/terrestrial) are conducted when necessary on a collective basis in restricted areas and time periods, with the organic insecticide spinosad being the one most widely applied in such cases.

**Figure 5:**
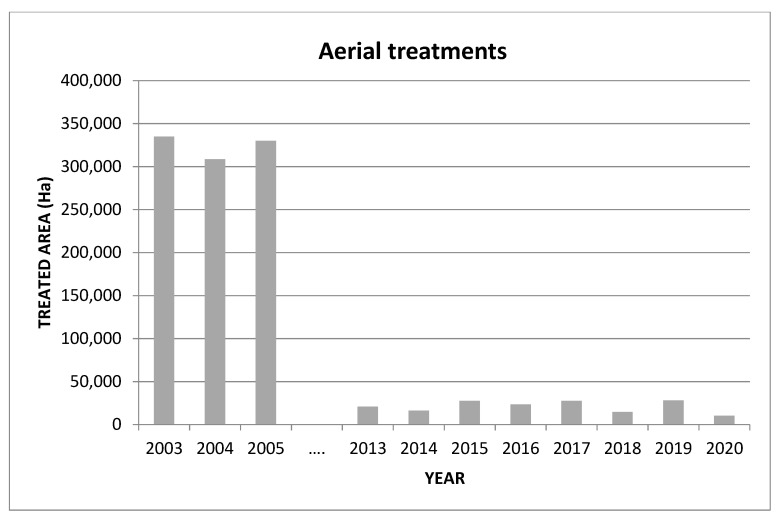
Area treated with insecticide bait sprays by aerial means from 2003 to present in the Valencian Community (Spain).

After the publication of European Directive 128/2009, a progressive withdrawal from the use of insecticides with the worst eco-toxicological profiles was initiated. After the implementation of the AW-IPM programme against *C. capitata* in the Valencian Community, the use of insecticides was significantly reduced. According to data from the Department of Agriculture of Valencia, more than 6 million liters of insecticides were sprayed by air per year at the beginning of this century, whereas in recent years the annual level has been reduced to only 150,000 L. The areas treated with insecticide sprays have also been significantly reduced (Figure 5). In addition, official data indicate that the trend for citrus exports and their economic importance is clearly positive for the Valencian Community, and that revenue has increased by more than 27% since 2005.

The success of the SIT essentially relies on the mating between released sterile males and wild females. This requires that sterile males be able to survive long enough in the field and successfully compete with wild males in finding and mating with wild females. A significant achievement during the last few years has been the development of genetic sexing strains for SIT programmes against *C. capitata* [21,28,31]. In order to assess the quality of mass-reared *C. capitata* flies, small- and large-scale field evaluations have been carried out [32,33,34,35]. In addition, many efforts are being made to enhance the AW-IPM programme, from improving the performance of sterile males to complementing the SIT with the use of biological control agents (EF and parasitoids) in order to reduce wild populations in the field.

Currently, citriculture in the Valencian Community is in a position to take a new, qualitative leap through widespread adoption of the AW-IPM approach. The adoption of AW-IPM for citrus has increased over the last few years [36], and it is important to promote its use and to continue with this positive trend. For that reason, it will be necessary to increase the dissemination of knowledge about the different strategies based on this system that can be used to control *C. capitata* with higher efficiency, using the SIT as the main control method in this region.

## Data Availability

The data presented in this study are available on request from the corresponding author.
